# Ultrastructural evaluation of lithium-induced autophagic and mitochondrial stress in 3D endometrial and neuroblastoma spheroids

**DOI:** 10.1038/s41598-025-21569-3

**Published:** 2025-10-28

**Authors:** Berna Yıldırım, Agnes Ansa Archibong-Omon, Ayhan Bilir

**Affiliations:** 1 Department of Histology and Embryology, Faculty of Medicine, İstanbul Atlas University, İstanbul, Turkey; 2 İstanbul Faculty of Medicine, Department of Histology and Embryology, İstanbul University, İstanbul, Turkey

**Keywords:** Mitophagy, Lithium chloride, 3D cancer spheroids, Endometrial cancer, Transmission electron microscopy, Biochemistry, Cancer, Cell biology

## Abstract

Lithium chloride (LiCl), a widely used mood stabilizer, has been reported to modulate selective autophagy pathways, including mitophagy. However, its ultrastructural effects in three-dimensional (3D) tumor models remain incompletely characterized. In this study, we examined the subcellular alterations induced by LiCl in 3D spheroid cultures derived from Ishikawa endometrial cancer and SH-SY5Y neuroblastoma cells. Spheroids were treated with 1, 10, or 50 mM LiCl and analyzed using transmission electron microscopy (TEM). The analysis revealed double-membrane-bound vesicles surrounding degenerating mitochondria, along with cytoplasmic vacuolization and membrane remodeling. These morphological features are suggestive of mitophagic activity, accompanied by stress-related ultrastructural remodeling. Although molecular validation (e.g., LC3B or PINK1/Parkin Western blotting) was not performed, the observed ultrastructural profiles are consistent with organelle-selective autophagy. These findings underscore the dose-dependent cellular responses to LiCl and support the value of 3D cancer spheroids as models to explore non-canonical autophagy-related stress pathways. Future studies incorporating molecular markers such as LC3B, PINK1, Parkin, and Lamin B1 will be essential to confirm these observations.

## Introduction

 Autophagy is a conserved cellular mechanism responsible for degrading dysfunctional organelles, misfolded proteins, and other cytoplasmic components via lysosomal pathways^1,2^. A selective form of autophagy, mitophagy, removes damaged mitochondria to maintain mitochondrial quality control and reduce cellular stress^3,4^.

Mitophagy involves multiple autophagy-related (Atg) proteins, particularly the Atg8/LC3 system^5^. LC3 (microtubule-associated protein 1 A/1B-light chain 3, MAP1LC3) exists as LC3-I in the cytosol and LC3-II on autophagosomal membranes^5^. The conversion of LC3-I to LC3-II and its interaction with mitochondrial receptors such as BNIP3, NIX, and FUNDC1 facilitate selective mitochondrial sequestration^6–8^. Transmission electron microscopy (TEM) remains the gold standard for detecting autophagic vesicles, particularly double-membrane-bound structures surrounding degenerating mitochondria^1,3^.

Lithium chloride (LiCl), widely used in psychiatry as a mood stabilizer, has attracted attention for its autophagy-modulating and anticancer properties^2,9,10^. Our previous studies demonstrated the cytotoxic and differentiation-modulating effects of LiCl in 2D endometrial and prostate cancer cell models, supporting its context-specific bioactivity in tumor systems^11^. LiCl acts by inhibiting inositol monophosphatase, thereby altering the IP3 pathway and indirectly affecting mTOR signaling^12–15^. Although increasing evidence links LiCl to autophagy and mitophagy, its ultrastructural effects, particularly in three-dimensional (3D) tumor models, remain underexplored. Our preclinical work in 2D endometrial models suggested a biphasic cytotoxic response to LiCl, but its subcellular remodeling effects in 3D cultures remained to be elucidated^16,17^.

3D spheroid models more closely mimic the tumor microenvironment than monolayer cultures, particularly in nutrient gradients, cell–cell communication, and stress responses. However, LiCl’s organelle-specific effects in these systems are still not well-defined. To address this, we used two tumor cell lines of different origin: Ishikawa endometrial adenocarcinoma and SH-SY5Y neuroblastoma cells^18^. SH-SY5Y is a widely used in vitro model for studying autophagy, particularly in the context of neurotoxicity and oxidative stress^19,20^. Recent studies show that LiCl can enhance mitophagy in SH-SY5Y cells through the PINK1/Parkin pathway and by inhibiting GSK-3β activity^4,18^.

By comparing these two cell lines, we aimed to assess whether LiCl induces organelle-specific subcellular remodeling, and whether such effects are cell type–dependent. This dual-model approach enhances the translational relevance of our findings and underscores the utility of 3D spheroids in visualizing autophagy-related stress responses at the ultrastructural level.

## Materials and methods

### Cell lines and chemicals

Human endometrial Ishikawa and neuroblastoma SH-SY5Y cells (American Type Culture Collection, Manassas, VA, USA) were maintained under standard culture conditions in RPMI 1640 medium (Biological Industries, Beit Haemek, Israel) supplemented with 10% fetal bovine serum (GIBCO, Invitrogen Co, Paisley, UK), 1% penicillin, and 100 µg/ml streptomycin (Sigma Co., St Louis, MO, USA). The cultures were maintained at 37 °C in a humidified incubator with 5% CO₂. Cells were passaged using Ca²⁺ and Mg²⁺-free phosphate-buffered saline (PBS) and 0.5% trypsin (Sigma Co., St Louis, MO, USA). Passages were carried out twice weekly, and semi-confluent cultures were used for all experiments. Final concentrations of lithium chloride (LiCl; Sigma-Aldrich Chemie GmbH) at 1, 10, and 50 mM were added to the cell cultures in equal volumes.

### Experimental design

The choice of LiCl concentrations (1, 10, and 50 mM) was guided by previous studies reporting its dose-dependent effects on autophagy and cytotoxicity. Specifically, 10 mM LiCl is considered an effective but tolerable concentration for mTOR-independent autophagy induction without overt cell death^13^. In contrast, 50 mM is associated with pronounced cellular stress, mitochondrial damage, and cytoplasmic degradation, representing a threshold for autophagy-related cell death^21,22^. Including 1 mM enabled us to observe potential early-stage or sub-threshold responses. While the employed concentrations exceed therapeutic plasma levels observed in patients (typically 0.6–1.2 mM)^23^, they are consistent with prior in vitro studies modeling lithium-induced autophagy and stress responses in cancer cells^13,24^.

The selected time points of 24 and 72 h were chosen to capture both early and late cellular responses to LiCl exposure. The 24-h time point allows the detection of rapid autophagic or proliferative responses, as acute BrdU (5-bromo-2′-deoxyuridine) incorporation changes have been reported within short exposures^25^. The 72-h time point provides a window to evaluate sustained or progressive effects, including cytotoxicity and viability loss; similar time-dependent LiCl–induced reductions in cell proliferation and viability have been observed in MM cells and myoblasts^26,27^. Supporting this, our own preliminary data demonstrate that 50 mM LiCl markedly decreased BrdU-positive cells at 72 h, indicating a strong inhibition of cellular proliferation under prolonged stress.

LiCl at 1, 10, and 50 mM concentrations was applied to both monolayer and spheroid cultures of Ishikawa and SH-SY5Y cells for 24 and 72 h. Cell proliferation, viability, and apoptosis indices were evaluated at both time points. Cellular ultrastructure was assessed by transmission electron microscopy (TEM) following LiCl exposure. All in vitro experiments were performed in at least three independent biological replicates (*n* = 3), unless otherwise specified.

### Cell proliferation assay

Cells were grown to semi-confluence, trypsinized (Sigma T4799), and counted using a hemocytometer. A 200 µL suspension containing 3 × 10⁵ cells was seeded into each well of a six-well plate (Nunc-Thermo Fisher Scientific) containing 3–5 mL of freshly prepared medium and incubated overnight at 37 °C with 5% CO₂. The next day, the medium was replaced with fresh medium containing increasing concentrations of LiCl. Cultures were maintained at 37 °C with 5% CO₂ for 24–96 h. Following incubation, cells were trypsinized, and total cell numbers were counted using a hemocytometer. The proliferation index and growth rates were determined by comparing cell counts after treatment.

### Trypan blue exclusion assay

Viable and non-viable cell counts were determined using the Trypan Blue exclusion method, based on the principle that live cells exclude the dye, while non-viable cells do not. Following treatment, the proportion of live versus dead cells was calculated based on dye exclusion in Ishikawa and SH-SY5Y cells. Viable cells displayed a clear cytoplasm, while non-viable cells were stained blue.

### Three-dimensional (Spheroid) cell culture

A 3D multicellular spheroid model was established using a liquid-overlay technique. Adherent Ishikawa and SH-SY5Y cells were cultured at 37 °C in RPMI-1640 medium supplemented with 10% heat-inactivated FBS and 1% penicillin-streptomycin. Semi-confluent monolayers were trypsinized to obtain a single-cell suspension. Spheroids were generated by seeding 1 × 10⁶ cells into 5 mL RPMI 1640 in six-well plates precoated with a thin agar layer (1:1 mixture of 3% agar (Difco, Franklin Lakes, NJ, USA) and medium). Medium was changed twice a week.

### Immunohistochemistry – Bromodeoxyuridine labelling index (BrdU-LI)

BrdU (5-bromo-2-deoxyuridine, 20 µM, 1:200 dilution) was added to the spheroid cultures during the last hour of incubation. Spheroids were removed and washed with PBS (pH 7.4), then fixed in 10% formaldehyde (Riedel-de Haen) for 24 h at 4 °C. Following fixation, specimens were dehydrated in graded ethanol (Merck 100983), cleared in toluene, embedded in paraffin (Kimetsan KIM-PNB/O1CP/040220), and sectioned at 5 μm. Sections were dewaxed in toluene for 30 min, rehydrated, and washed with PBS. Antigen retrieval was performed with 3% trypsin at 37 °C for 30 min. Endogenous peroxidase activity was blocked with 0.5% H₂O₂. Sections were incubated with primary mouse anti-BrdU antibody (Lab Vision, UK), followed by biotinylated IgG and streptavidin-peroxidase conjugate (Lab Vision, UK). Visualization was achieved using AEC chromogen and counterstaining with Mayer’s hematoxylin. BrdU-positive cells displayed dark red nuclear staining, while negative cells were identified by blue hematoxylin or pale brownish nuclei. Approximately 900 cells were evaluated per group (3 spheroids × ~300 nuclei) by a blinded observer.

### Electron microscopic evaluation

Spheroids reaching 120–400 μm in diameter were used for electron microscopy. After LiCl treatment, spheroids were fixed in 2.5% glutaraldehyde in 0.1 M sodium cacodylate buffer and post-fixed in 1% osmium tetroxide for two hours. After uranyl acetate staining (1%), samples were embedded in Epon 812. Ultrathin sections were prepared, stained with Reynold’s lead citrate, and examined using a JEOL-JEM 1011 transmission electron microscope (JEOL Ltd., Japan). Ultrastructural images were acquired at 80 kV using a JEOL-JEM 1011 transmission electron microscope, typically at magnifications between 10,000× and 30,000×.

### Statistical analysis

All statistical analyses were performed using GraphPad Prism version 9 (GraphPad Software, San Diego, CA, USA). Data are presented as mean ± standard deviation (SD). For comparisons involving multiple groups with a single independent variable (e.g., different LiCl concentrations at a single time point), one-way ANOVA followed by Dunnett’s post hoc test was applied to compare each treatment group against the control. In experiments involving two independent variables (e.g., LiCl concentration and incubation time), two-way ANOVA was used, again followed by Dunnett’s multiple comparisons test versus control. When data did not meet normality assumptions, the Kruskal–Wallis test followed by Dunn’s post hoc test was performed. A p-value ≤ 0.05 was considered statistically significant.

### Compliance and ethics

No ethical approval was required for this study as only commercially available human cell lines were used.

## Results

### Spheroid morphology and growth kinetics

**Fig. 1:**
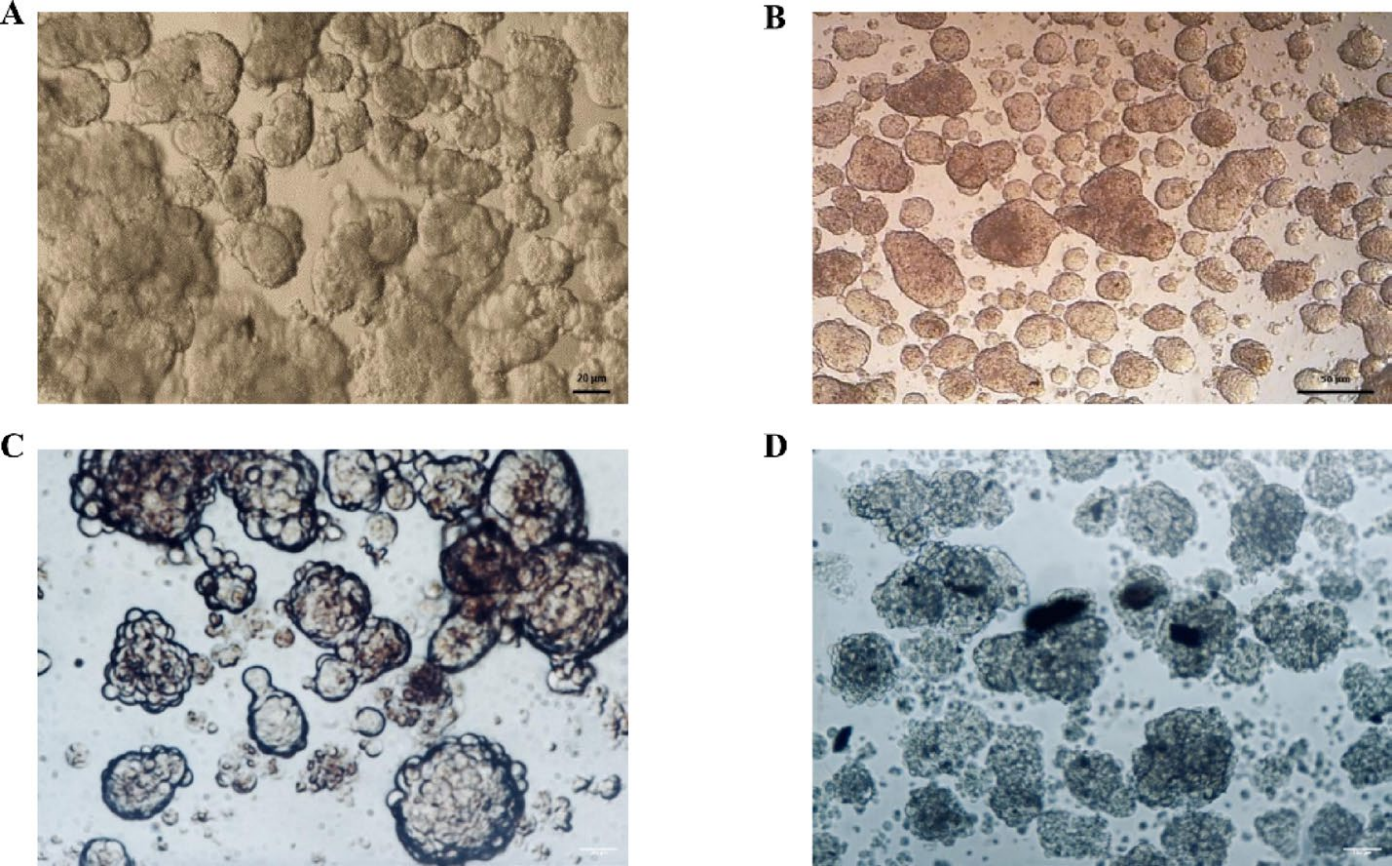
Morphology of control and LiCl-treated spheroids. (**A**, **B**) Representative inverted light microscopy images of untreated spheroids formed by Ishikawa (**A**) and SH-SY5Y (**B**) cells, both showing compact and cohesive structures under control conditions. Scale bars: **A** = 20 μm; **B** = 50 μm. (**C**, **D**) Spheroids treated with 10 mM LiCl for 24 h. Ishikawa (**C**) and SH-SY5Y (**D**) spheroids appeared irregular and fragmented with impaired borders, indicating concentration-dependent structural stress. Scale bars: **C** = 100 μm; **D** = 100 μm.

Ishikawa and SH-SY5Y cells were cultured in 3D non-adhesive spheroid systems using the agar-overlay technique and subsequently treated with increasing concentrations of LiCl. Under control conditions, both cell lines formed compact, rounded, and cohesive spheroids (Fig. 1A–B). In contrast, exposure to 10 mM LiCl resulted in irregular and fragmented spheroids with impaired borders and disrupted architecture (Fig. 1C–D). These alterations were further accentuated at higher concentrations such as 50 mM, as demonstrated in later TEM analyses.

### Cell proliferation in monolayer cultures

**Fig. 2:**
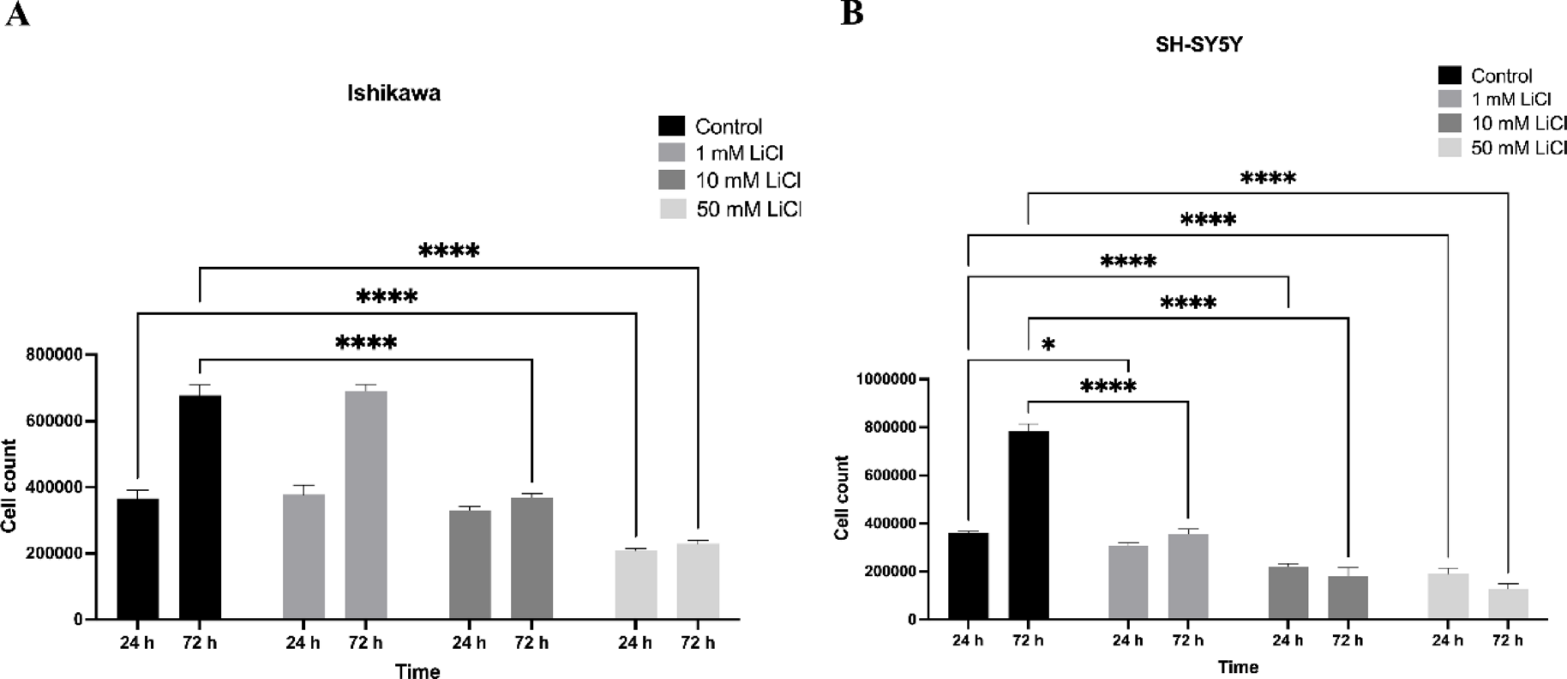
Lithium suppresses proliferation in Ishikawa and SH-SY5Y monolayers. (**A**) Cell counts of Ishikawa cells treated with LiCl (1–50 mM) for 24–72 h. (**B**) Cell counts of SH-SY5Y cells under the same conditions. Data are mean ± SD from three independent experiments; *p < 0.05, **p < 0.01, ***p < 0.001, ****p < 0.0001.

Cell proliferation was evaluated in Ishikawa and SH-SY5Y monolayers treated with LiCl (1–50 mM) for 24 and 72 h. A dose- and time-dependent reduction in cell counts was observed in both cell types, with the effect being more pronounced in SH-SY5Y cells. At 50 mM and 72 h, SH-SY5Y proliferation was almost completely suppressed (*p* < 0.01), whereas Ishikawa cells showed a milder but still significant decline compared with controls (Fig. 2A–B). These results indicate that SH-SY5Y monolayers are more sensitive to LiCl exposure than Ishikawa cells.

These 2D monolayer assays were conducted to determine cytotoxic thresholds and optimize dose–time windows for downstream 3D spheroid experiments. By comparing epithelial (Ishikawa) and neuronal (SH-SY5Y) lineages, we established baseline sensitivities and ensured that subsequent 3D analyses would be performed under non-lethal, but stress-inducing, conditions.

### BrdU labeling reveals proliferation suppression in spheroids

**Fig. 3:**
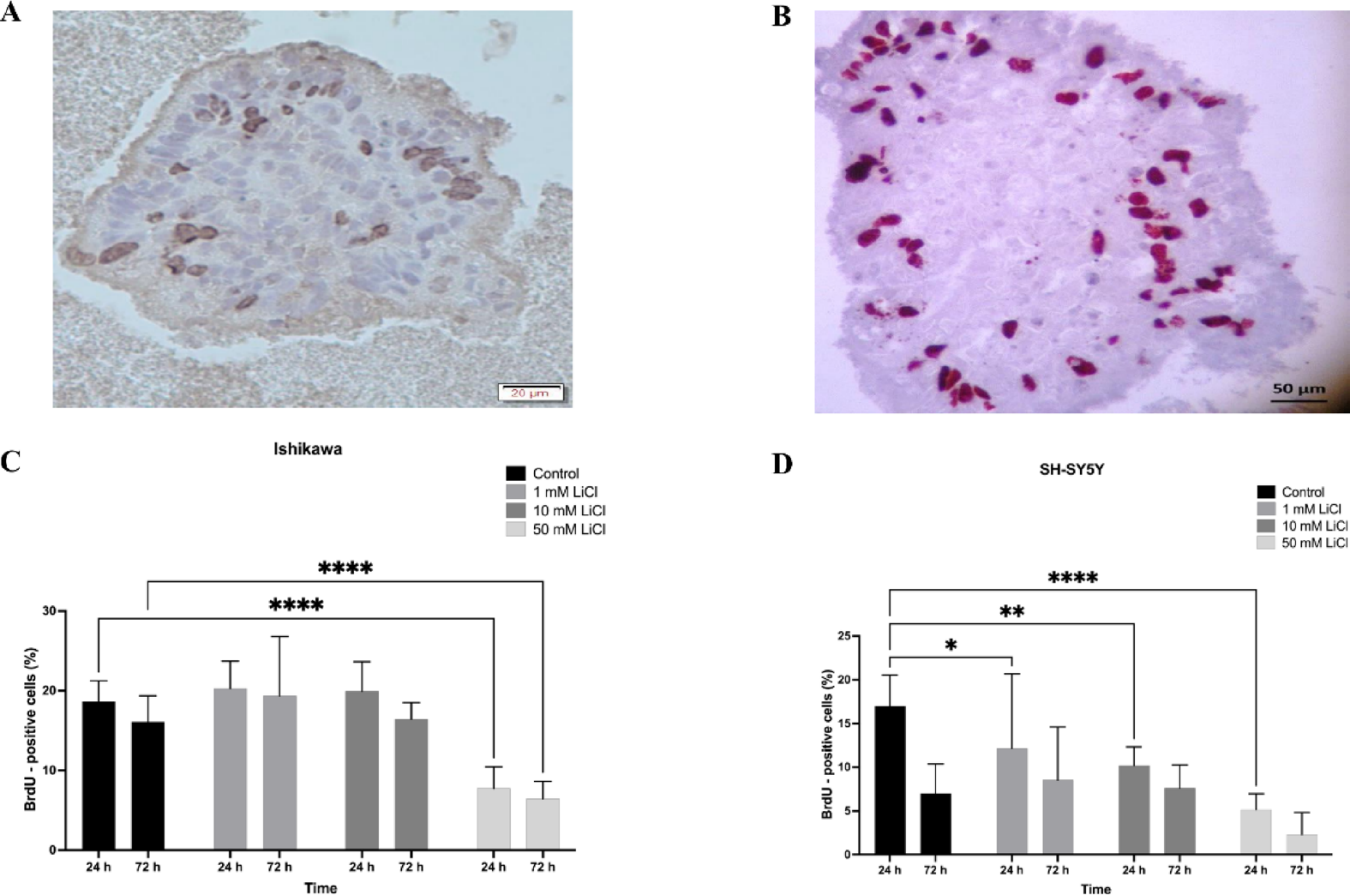
LiCl suppresses proliferation in 3D Ishikawa and SH-SY5Y spheroids. (**A**, **B**) Representative BrdU immunohistochemistry images showing S-phase nuclei (brown) in Ishikawa (**A**) and SH-SY5Y (**B**) spheroids. Scale bars: **A** = 20 μm; **B** = 50 μm. (**C**, **D**) Quantification of BrdU-positive cell fractions in Ishikawa (**C**) and SH-SY5Y (**D**) spheroids after LiCl treatment (1–50 mM, 24–72 h), demonstrating dose- and time-dependent reduction in proliferation. Data are mean ± SD from three independent experiments; *p < 0.05, **p < 0.01, ***p < 0.001, ****p < 0.0001.

BrdU immunolabeling was performed to evaluate S-phase cell fractions in Ishikawa and SH-SY5Y spheroids after LiCl treatment (1–50 mM) for 24 and 72 h. A significant, dose-dependent reduction in BrdU-positive nuclei was observed in both models, with the most marked suppression at 50 mM and 72 h. Quantitative analysis confirmed a strong antiproliferative effect of LiCl in both epithelial and neuronal spheroids (Fig. 3C–D).

### Cell viability in spheroids

**Fig. 4:**
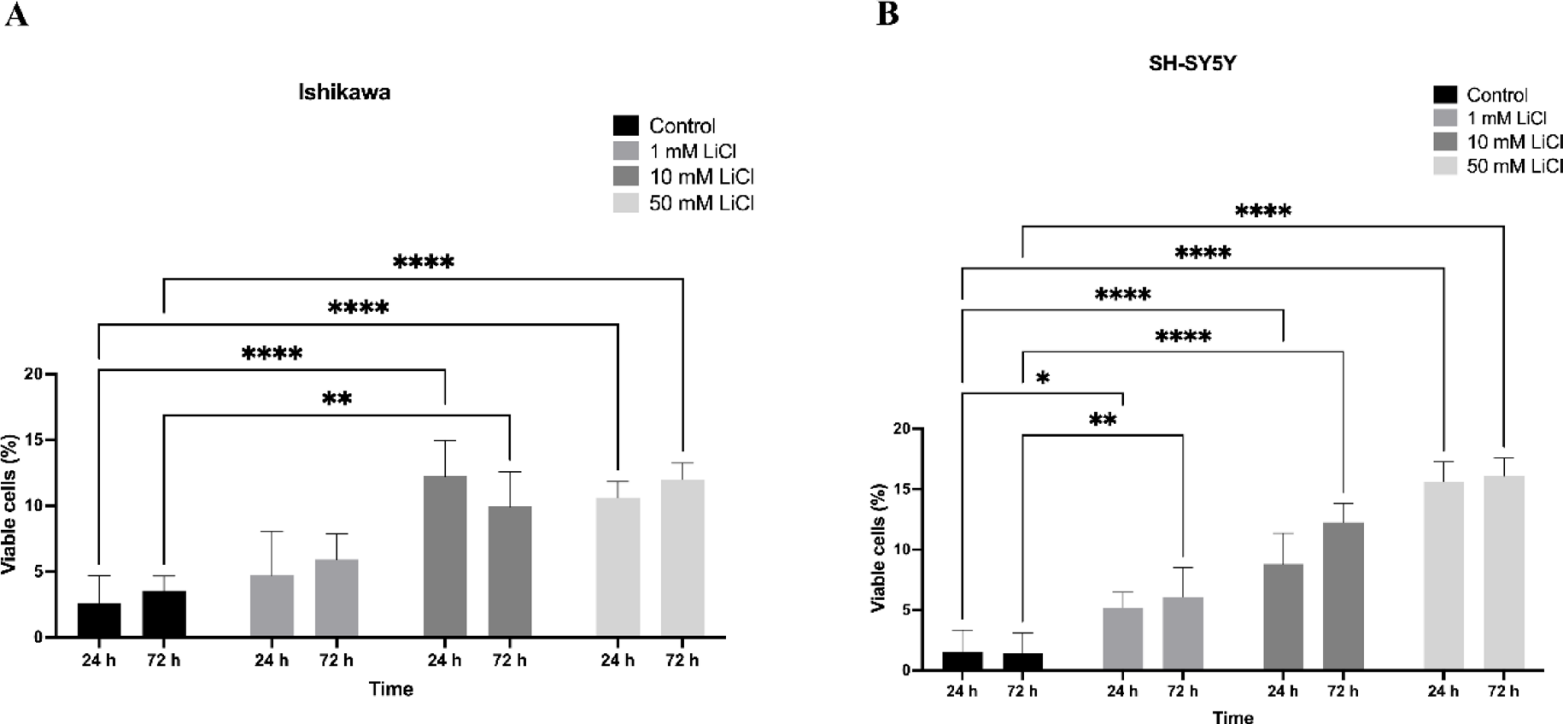
LiCl reduces spheroid cell viability. Trypan Blue exclusion assay showing viability of Ishikawa (left) and SH-SY5Y (right) spheroids after LiCl treatment (1, 10, and 50 mM; 24–72 h). Dose- and time-dependent decreases in viable cell percentages were observed in both models, with SH-SY5Y spheroids being more sensitive at higher concentrations. Data are mean ± SD from three independent experiments; *p < 0.05, **p < 0.01, ***p < 0.001, ****p < 0.0001.

Cell viability in Ishikawa and SH-SY5Y spheroids was assessed by Trypan Blue exclusion after LiCl treatment (1–50 mM, 24–72 h). Viable cell percentages decreased in a dose- and time-dependent manner, with SH-SY5Y spheroids showing greater sensitivity, especially at higher doses. These viability results paralleled the proliferation data, confirming a consistent cytotoxic effect of LiCl across both models (Fig. 4). Although colorimetric assays such as CCK-8 or MTT could provide more sensitive viability measurements, the Trypan Blue exclusion method was chosen for its rapid and cost-effective assessment of membrane integrity across multiple dose-time groups. Future studies may incorporate CCK-8 or similar assays for enhanced quantitative precision.

### Ultrastructural analysis of control spheroids (TEM)

**Fig. 5:**
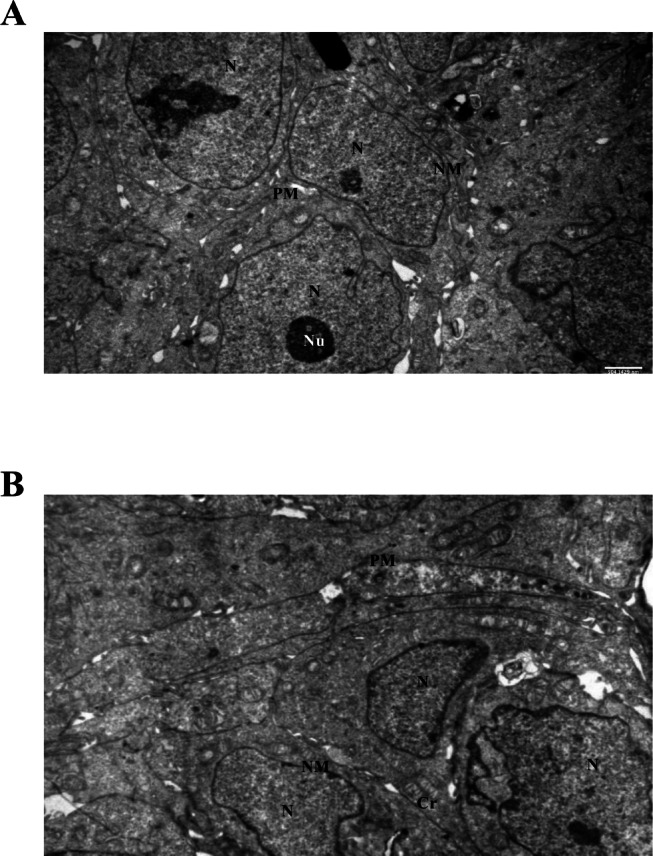
Control Ishikawa spheroid ultrastructure by TEM. Representative TEM images of untreated spheroids at 24 h (**A**) and 72 h (**B**) showing intact nuclei (N), nucleoli (Nu), mitochondria (Cr), and well-defined nuclear (NM) and plasma membranes (PM). Scale bars: 500 nm. Abbreviations: N, nucleus; Nu, nucleolus; NM, nuclear membrane; PM, plasma membrane; Cr, cristae.

Transmission electron microscopy (TEM) of untreated Ishikawa spheroids revealed well-preserved cellular ultrastructure at both 24 and 72 h. Mitochondria exhibited intact cristae, nuclei and nucleoli were clearly visible, and both nuclear and plasma membranes remained unaltered. These features serve as a baseline for subsequent comparisons with LiCl-treated spheroids (Fig. 5A–B).

### Mitochondrial alterations and mitophagy-like remodeling in LiCl-treated Ishikawa spheroids

**Fig. 6:**
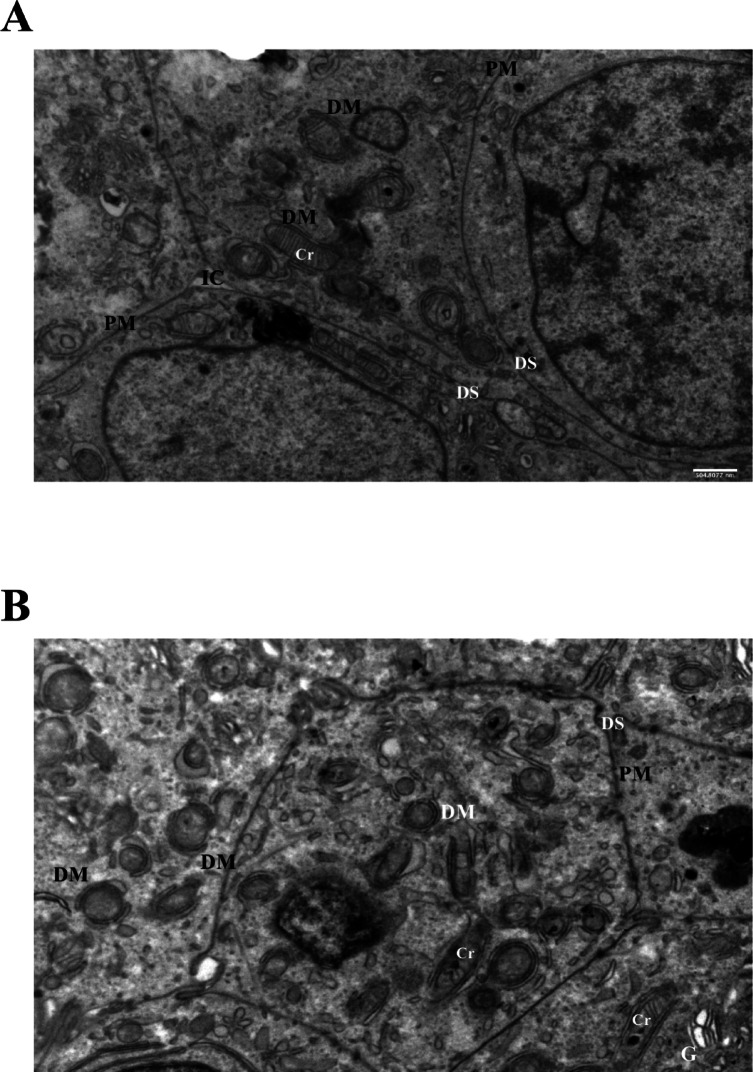
Mitochondrial injury and mitophagy in LiCl-treated Ishikawa spheroids. (**A**) 1 mM LiCl (72 h): mild mitochondrial changes, early vacuolization, and initial cristae loss. (**B**) 10 mM LiCl (72 h): pronounced mitochondrial damage, double-membrane structures (DM), and intercellular disorganization. Although mitophagy-related markers (LC3-II, Atg proteins) were not analyzed, the morphology is consistent with mitophagy initiation. Scale bars: 500 nm. Abbreviations: DM, double membrane; Cr, cristae; PM, plasma membrane; IC, intercellular space; DS, desmosome; G, Golgi.

LiCl treatment induced progressive mitochondrial injury and autophagic remodeling in Ishikawa spheroids. At 1 mM LiCl (72 h), mild mitochondrial swelling and initial cristae loss were detected (Fig. 6A). At 10 mM LiCl (72 h), mitochondria showed pronounced damage and were frequently surrounded by double-membrane structures, morphologically consistent with isolation membranes described in mitophagy-like processes. Although markers such as LC3-II or Atg proteins were not analyzed, the observed ultrastructure is suggestive of mitophagic remodeling, but without molecular validation, these findings should be interpreted as mitophagy-like rather than definitive evidence of mitophagy. Intercellular disorganization and partial cytoskeletal disruption became more evident at 10 mM (Fig. 6B). Despite these changes, desmosomal junctions remained visible, indicating that some cell–cell contacts persisted.

### Dose-dependent cytoplasmic lysis and organelle remodeling

**Fig. 7:**
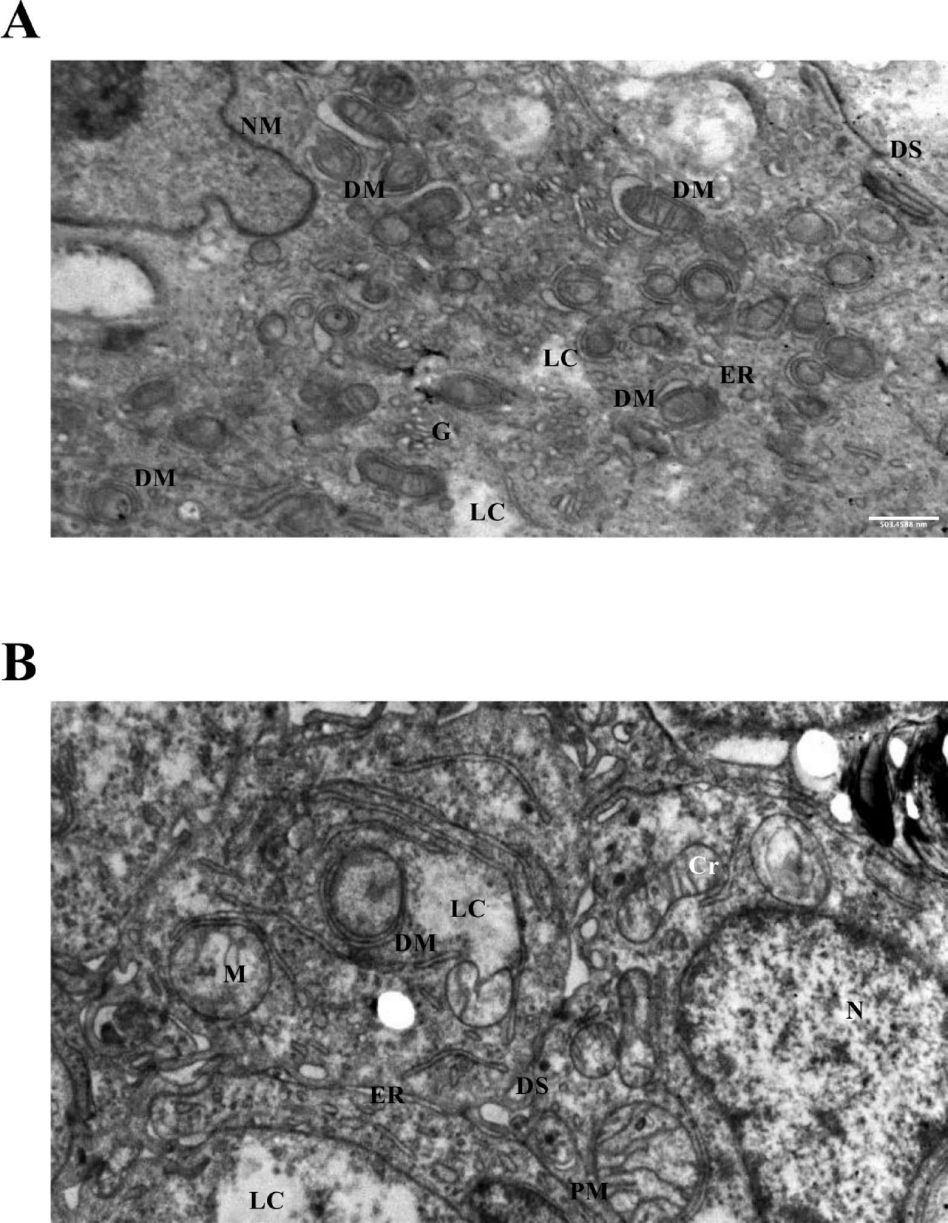
TEM images of Ishikawa spheroids after high-dose LiCl exposure. (**A**) Treatment with 50 mM LiCl for 24 h resulted in mitochondrial swelling and the appearance of double-membrane-bound vesicles (DM), consistent with early autophagic remodeling. (**B**) After 72 h of 50 mM LiCl exposure, cells exhibited severe mitochondrial degeneration, extensive cytoplasmic lysis (LC), dilated endoplasmic reticulum (ER), enlarged Golgi apparatus (G), and electron-dense aggregates.Scale bars: 500 nm.Abbreviations: DM, double membrane; LC, lytic cytoplasm; ER, endoplasmic reticulum; G, Golgi apparatus; N, nucleus; NM, nuclear membrane; DS, desmosome; Cr, cristae; M, mitochondria.

Prolonged exposure to 50 mM LiCl for 24 and 72 h resulted in pronounced ultrastructural changes in Ishikawa spheroids. At 24 h, mitochondrial swelling and the formation of double-membrane-bound vesicles were observed (Fig. 7A), suggesting the initiation of mitophagy-like processes. By 72 h, severe mitochondrial degeneration, cytoplasmic lysis, dilation of the endoplasmic reticulum (ER), expansion of the Golgi apparatus, and the presence of electron-dense aggregates, possibly intermediate filaments or protein accumulations, became prominent (Fig. 7B). These findings indicate that high-dose lithium treatment disrupts multiple organellar systems, leading to advanced autophagic stress and loss of cellular integrity in epithelial spheroids.

### Selective nuclear envelope breakdown in SH-SY5Y spheroids

**Fig. 8:**
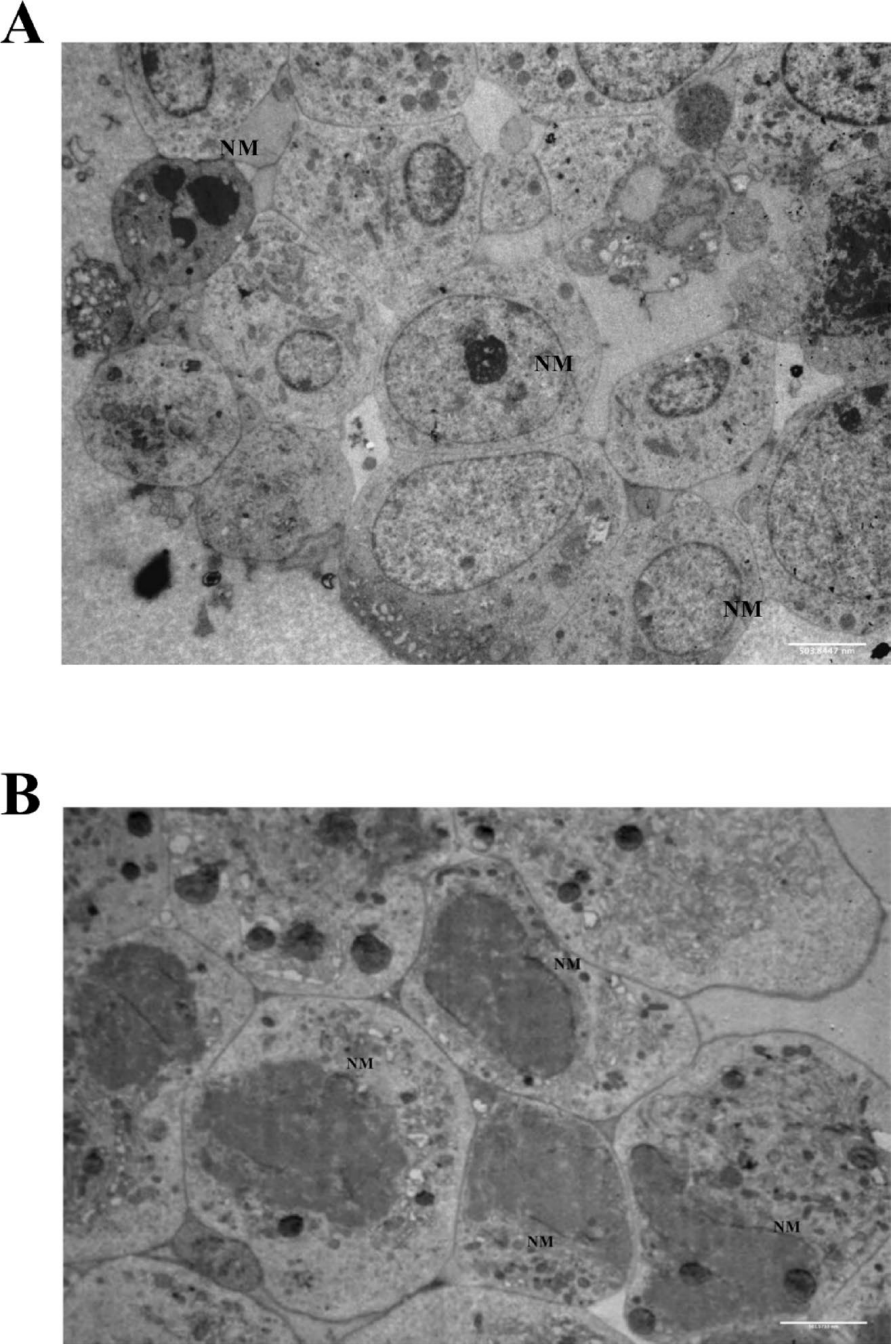
TEM analysis of SH-SY5Y spheroids: control vs. low-dose LiCl. (**A**) Control SH-SY5Y spheroid cell showing intact nuclear membranes (NM). (**B**) SH-SY5Y spheroid cell treated with 1 mM LiCl for 72 h displaying localized nuclear envelope breakdown (arrow) and residual nuclear membrane fragments, while cytoplasmic membranes remain preserved. Scale bar: 500 nm. Abbreviation: NM, nuclear membrane.

TEM analysis of untreated SH-SY5Y spheroids revealed intact nuclear membranes and well-preserved cellular ultrastructure (Fig. 8A). Upon treatment with 1 mM LiCl for 72 h, focal breakdown of the nuclear envelope was observed, with visible residual nuclear membrane fragments and otherwise preserved cytoplasmic membranes (Fig. 8B). These findings indicate a localized nuclear stress response to lithium exposure, which may represent early nucleophagy or a cell cycle related event. Further studies incorporating markers such as Cyclin B or CDC2 will be necessary to confirm the mechanistic basis of this selective nuclear remodeling.

## Discussion

Autophagy is an evolutionarily conserved catabolic process that facilitates the degradation of intracellular components via lysosomes, thereby maintaining cellular homeostasis^1,2^. Mitophagy, a selective form of autophagy, specifically targets dysfunctional mitochondria for lysosomal degradation and plays a key role in mitochondrial quality control^3,21^. This process involves receptor proteins such as BNIP3, NIX, and FUNDC1, which interact with LC3 through their LC3-interacting regions (LIR motifs) to initiate mitophagosome formation^6,14,22,28^. Given the critical role of mitophagy in regulating stress responses and cell fate, understanding how pharmacological agents such as lithium chloride (LiCl) influence this process is of increasing interest.

In Ishikawa spheroids, we detected double-membrane autophagosomes containing damaged mitochondria following LiCl treatment. These structures are morphologically compatible with mitophagy-like remodeling, as described in previous ultrastructural studies^6,14,29–31^. In the present study, however, mitophagy-like changes appeared as a secondary response, while nuclear envelope remodeling and nucleophagy predominated under similar conditions.

Although the primary focus of this study was on endometrial cancer spheroids, additional experiments in SH-SY5Y spheroids were performed to assess the generalizability of LiCl-induced nuclear alterations. In this model, high-dose LiCl caused nuclear envelope disintegration and partial cytoplasmic dissolution. These changes may reflect nuclear stress responses such as nucleophagy or mitotic catastrophe; however, without molecular validation using markers such as Lamin B1 or CDK1/Cyclin B, the interpretation remains preliminary^31^. Importantly, nuclear envelope elongation and sequestration were also observed, features that are morphologically consistent with nucleophagy as described in previous reports, although further molecular confirmation will be required.

LiCl exerts dual, dose- and context-dependent effects. At low concentrations, it can induce mitophagy or adaptive autophagy, whereas higher doses trigger cytotoxic responses^9,12,13,24,31^. Our observation of a biphasic effect on proliferation is consistent with previous studies in which low-dose LiCl transiently promoted survival, while higher concentrations induced cytotoxicity. Comparable dose-dependent patterns have been reported in prostate, breast, and endometrial cancer models, underscoring the need for careful dose calibration in therapeutic contexts^9,10,32^.

LiCl also modulates the Wnt/β-Catenin pathway, which influences stemness and proliferation^33–36^. In our models, however, BrdU incorporation and cell proliferation were suppressed at 50 mM in both 2D and 3D cultures, indicating a cytotoxic shift. Reduced BrdU labeling suggests impaired DNA synthesis, although additional assays (e.g., cleaved caspase-3 or cell cycle analysis) would be required to determine whether this reflects proliferative arrest or cell death. Interestingly, high-dose LiCl induced mitophagic structures while simultaneously suppressing proliferation. This paradox may reflect excessive mitochondrial degradation culminating in a bioenergetic collapse. Prior reports have similarly noted that excessive mitophagy can impair ATP production and promote senescence or death rather than maintaining homeostasis^6,21,37,38^.

Dose selection in this study was guided by preliminary sensitivity testing. SH-SY5Y spheroids were treated with 1 mM LiCl, as higher doses caused severe cytotoxicity and impaired spheroid formation. In contrast, Ishikawa spheroids tolerated higher concentrations and were therefore analyzed at 1, 10, and 50 mM for 72 h, enabling assessment of dose-dependent ultrastructural changes. This approach highlights the distinct pharmacodynamic responses of epithelial versus neuronal cells in 3D models.

Although we observed ultrastructural evidence of mitophagy, molecular confirmation using LC3-II, BNIP3, NIX, or FUNDC1 was not performed. Nonetheless, TEM analyses across a dose range (1, 10, and 50 mM) revealed a progressive pattern of mitochondrial injury and autophagosome formation, consistent with dose-dependent initiation of mitophagy-like processes. This underscores the value of multi-dose designs in ultrastructural studies, as single-dose analyses may overlook gradual organelle-specific stress responses.

Dose–time combinations were strategically selected (e.g., 1 and 10 mM for Ishikawa, 1 mM for SH-SY5Y) based on viability and proliferation data to capture peak morphological changes. This approach enabled us to visualize representative ultrastructural alterations without introducing redundancy. Importantly, Ishikawa spheroids tolerated higher doses than SH-SY5Y spheroids, which rapidly disintegrated at concentrations above 1 mM, highlighting lineage-specific sensitivity.

The use of 3D spheroids instead of conventional monolayers constitutes a major methodological strength. Spheroids preserve gradients of nutrients, oxygen, and drug penetration, as well as cell–cell interactions, features that more closely resemble in vivo tumors^39,40^. Consequently, the ultrastructural changes observed here may better approximate in vivo-like stress responses to lithium. Few prior studies have explored mitophagy in endometrial cancer spheroids, further underscoring the novelty of our model.

Electron-dense filament-like structures were occasionally noted adjacent to mitophagosomes. While these morphologically resemble intermediate filaments, their role in lithium-induced stress remains uncertain and was not the primary focus of this study.

Lithium differs from classical mitophagy inducers such as CCCP, oligomycin, or antimycin A, which act primarily through mitochondrial depolarization or respiratory complex inhibition^41,42^. In contrast, LiCl induced ultrastructural signatures of selective mitophagy and nucleophagy at lower concentrations (1–10 mM), while at 50 mM, severe cytoplasmic lysis and nuclear disintegration predominated. These findings suggest that lithium’s effects diverge from canonical uncouplers, combining modulatory and cytotoxic features in a dose-dependent manner.

In this context, ‘cytoplasmic lysis’ refers to vacuolar disintegration and membrane remodeling characteristic of advanced autophagic stress, rather than necrosis. These observations support the idea that LiCl can act as both an autophagy modulator and a cytotoxic agent, depending on cell type and dose. However, the highest concentration used (50 mM) exceeds physiological levels, limiting direct clinical extrapolation. Future work should explore subtoxic, physiologically relevant doses.

Finally, we acknowledge that our interpretations of mitophagy are based exclusively on ultrastructural hallmarks—double-membraned vesicles enclosing degenerating mitochondria—which are morphologically compatible with mitophagy-like remodeling, as described in previous ultrastructural studies^6,14^. No molecular markers such as LC3, BNIP3, or PINK1/Parkin were examined; therefore, the activation of canonical mitophagy or nucleophagy pathways cannot be definitively confirmed in this study and should be regarded as descriptive rather than definitive. Despite this limitation, our findings indicate that LiCl induces organelle-specific stress responses, including both mitophagy-like and nuclear changes, in a dose- and cell type–dependent manner. Validation by Western blotting and immunofluorescence will be essential in future studies.

## Conclusion

This study demonstrates that lithium chloride (LiCl) induces ultrastructural features of organelle-specific stress in a dose- and cell type–dependent manner. In Ishikawa endometrial cancer spheroids, LiCl exposure led to the formation of double-membrane-bound vesicles enclosing degenerating mitochondria, compatible with mitophagy-like processes. In contrast, SH-SY5Y neuroblastoma spheroids displayed focal nuclear envelope disintegration and sequestration, suggestive of a nucleophagic response. Together, these findings highlight the dual impact of lithium on both mitochondrial and nuclear compartments.

It must be emphasized that these interpretations rely exclusively on transmission electron microscopy (TEM). No molecular markers such as LC3, BNIP3, or PINK1/Parkin were examined, and therefore the precise activation of mitophagy or nucleophagy pathways remains to be validated. In addition, the highest concentration applied (50 mM) exceeds physiological lithium exposure, limiting direct therapeutic extrapolation. Thus, our interpretation of mitophagy is descriptive, based on morphological criteria, and should not be taken as definitive evidence of pathway activation.

Despite these limitations, our work underscores two key contributions. First, it highlights the utility of 3D spheroid models combined with TEM in capturing subtle, non-canonical forms of autophagy that may be missed in 2D systems. Second, it reveals that LiCl can exert context-dependent effects, acting as an autophagy modulator at lower doses while provoking severe nuclear and cytoplasmic stress at higher doses. Future studies integrating physiologically relevant dose ranges with molecular assays (Western blotting, immunofluorescence, functional autophagy flux measurements) will be essential to confirm these pathways and clarify lithium’s dual role in regulating cellular homeostasis and stress responses in tumor systems.

## Data Availability

The data supporting the findings of this study are included in the manuscript and its figures. Additional data are available from the corresponding author upon reasonable request.

## References

[1] Mizushima, N. & Komatsu, M. Autophagy: renovation of cells and tissues. *Cell* **147**, 728–741 (2011). 22078875 10.1016/j.cell.2011.10.026

[2] Zaffagnini, G. & Martens, S. Mechanisms of selective autophagy. *J. Mol. Biol.* **428**, 1714–1724 (2016). 26876603 10.1016/j.jmb.2016.02.004 PMC4871809

[3] Kim, I., Rodriguez-Enriquez, S. & Lemasters, J. J. Selective degradation of mitochondria by mitophagy. *Arch. Biochem. Biophys.* **462**, 245–253 (2007). 17475204 10.1016/j.abb.2007.03.034 PMC2756107

[4] Ma, W. et al. Targeting selective autophagy and beyond: from underlying mechanisms to potential therapies. *J. Adv. Res.* **65**, 297–327 (2024). 38750694 10.1016/j.jare.2024.05.009 PMC11518956

[5] Lee, Y. K. & Lee, J. A. Role of the mammalian ATG8/LC3 family in autophagy: differential and compensatory roles in the Spatiotemporal regulation of autophagy. *BMB Rep.* **49**, 424–430 (2016). 27418283 10.5483/BMBRep.2016.49.8.081 PMC5070729

[6] Feng, D., Liu, L., Zhu, Y. & Chen, Q. Molecular signaling toward mitophagy and its physiological significance. *Exp. Cell. Res.* **319**, 1697–1705 (2013). 23603281 10.1016/j.yexcr.2013.03.034

[7] Gómez-Virgilio, L. et al. Autophagy: A key regulator of homeostasis and disease: an overview of molecular mechanisms and modulators. *Cells* **11**, 2262 (2022). 35892559 10.3390/cells11152262 PMC9329718

[8] Liu, L. et al. Mitochondrial outer-membrane protein FUNDC1 mediates hypoxia-induced mitophagy in mammalian cells. *Nat. Cell. Biol.* **14**, 177–185 (2012). 22267086 10.1038/ncb2422

[9] Erguven, M., Oktem, G., Kara, A. N. & Bilir, A. Lithium chloride has a biphasic effect on prostate cancer stem cells and a proportional effect on midkine levels. *Oncol. Lett. 12 4 2948–2955*. 10.3892/ol.2016.4946 PMC5038888 27703531

[10] Eren, S. N. et al. Lithium chloride potentiates vinorelbine and acetaminophen cytotoxicity in MDAH-2774 cell line in vitro.* Medical Res. Arch.*** 6** (11) (2018).

[11] Erguven, M., Oktem, G., Kara, A. N. & Bilir, A. Lithium chloride has a biphasic effect on prostate cancer stem cells and a proportional effect on midkine levels. *Oncol. Lett.* **12**, 2948–2955 (2016). 27703531 10.3892/ol.2016.4946 PMC5038888

[12] Sarkar, S., Rubinsztein, D. C. & Inositol IP3 levels regulate autophagy: biology and therapeutic speculations. *Autophagy* **2**, 132–134 (2006). 16874097 10.4161/auto.2387

[13] Sarkar, S. et al. Lithium induces autophagy by inhibiting inositol monophosphatase. *J. Cell. Biol.* **170**, 1101–1111 (2005). 16186256 10.1083/jcb.200504035 PMC2171537

[14] Chourasia, A. H., Boland, M. L. & Macleod, K. F. Mitophagy and cancer. *Cancer Metab.* **3**, 4 (2015). 25810907 10.1186/s40170-015-0130-8 PMC4373087

[15] Snitow, M. E., Bhansali, R. S. & Klein, P. S. Lithium and therapeutic targeting of GSK-3. *Cells* **10**, 255 (2021). 33525562 10.3390/cells10020255 PMC7910927

[16] Tural, E. et al. The effects of lithium, Metformin and everolimus substances on cell growth in 2D and 3D Ishikawa endometrial carcinoma cell culture. *Pamukkale Med. J.* **17**, 560–576 (2024).

[17] Bilir, A., Aynacioglu, A. S. & Tuna, M. Y. The possible interactions and therapeutic roles of lithium chloride and midkine on cancer treatment. *Crit. Rev. Oncog.* **24**, 35–45 (2019). 31679218 10.1615/CritRevOncog.2018029410

[18] Hou, L. et al. Lithium protects dopaminergic cells from rotenone toxicity via autophagy enhancement. *BMC Neurosci.* **16**, 82 (2015). 26608648 10.1186/s12868-015-0222-y PMC4658766

[19] Park, S. Y. & Koh, H. C. FUNDC1 regulates receptor-mediated mitophagy independently of the PINK1/Parkin-dependent pathway in rotenone-treated SH-SY5Y cells. *Food Chem. Toxicol.* **137**, 111163 (2020). 32001317 10.1016/j.fct.2020.111163

[20] Wang, X. et al. [Makale başlığı]. *Front Mol. Neurosci*. **17**, 1359294. 10.3389/fnmol.2024.1359294 (2024). 10.3389/fnmol.2024.1359294 PMC11066238 38706874

[21] Ashrafi, G. & Schwarz, T. L. The pathways of mitophagy for quality control and clearance of mitochondria. *Cell. Death Differ.* **20**, 31–42 (2013). 22743996 10.1038/cdd.2012.81 PMC3524633

[22] Kanki, T. Nix, a receptor protein for mitophagy in mammals. *Autophagy* **6**, 433–435 (2010). 20200478 10.4161/auto.6.3.11420

[23] Chiu, C. T., Wang, Z., Hunsberger, J. G. & Chuang, D. M. Therapeutic potential of mood stabilizers lithium and valproic acid: beyond bipolar disorder. *Pharmacol. Rev.* **65**, 105–142 (2013). 23300133 10.1124/pr.111.005512 PMC3565922

[24] Motoi, Y., Shimada, K., Ishiguro, K. & Hattori, N. Lithium and autophagy. *ACS Chem. Neurosci.* **5**, 434–442 (2014). 24738557 10.1021/cn500056q PMC4063500

[25] Zanni, G. et al. Lithium increases proliferation of hippocampal neural stem/progenitor cells and rescues irradiation-induced cell cycle arrest in vitro. *Oncotarget* **6**, 37083–37097 (2015). 26397227 10.18632/oncotarget.5191 PMC4741917

[26] Yao, R. et al. Lithium chloride inhibits cell survival, overcomes drug resistance, and triggers apoptosis in multiple myeloma via activation of the Wnt/β-catenin pathway. *Am. J. Transl Res.* **10**, 2610–2618 (2018). 30210697 PMC6129537

[27] Lee, J. H. et al. Lithium chloride protects against sepsis-induced skeletal muscle atrophy and cancer cachexia. *Cells* **10**, 1017 (2021). 33925786 10.3390/cells10051017 PMC8146089

[28] Liu, L., Sakakibara, K., Chen, Q. & Okamoto, K. Receptor-mediated mitophagy in yeast and mammalian systems. *Cell. Res.* **24**, 787–795 (2014). 24903109 10.1038/cr.2014.75 PMC4085769

[29] J Klionsky, D. et al. Guidelines for the use and interpretation of assays for monitoring autophagy (4th edition)1. *Autophagy* **17**, 1–382 (2021). 33634751 10.1080/15548627.2020.1797280 PMC7996087

[30] Sun, N. et al. Measuring in vivo mitophagy. *Mol. Cell.* **60**, 685–696 (2015). 26549682 10.1016/j.molcel.2015.10.009 PMC4656081

[31] Ryves, W. J., Dalton, E. C., Harwood, A. J. & Williams, R. S. GSK-3 activity in neocortical cells is inhibited by lithium but not carbamazepine or valproic acid. *Bipolar Disord*. **7**, 260–265 (2005). 15898963 10.1111/j.1399-5618.2005.00194.x PMC1249491

[32] Suganthi, M., Sangeetha, G., Gayathri, G. & Ravi Sankar, B. Biphasic dose-dependent effect of lithium chloride on survival of human hormone-dependent breast cancer cells (MCF-7). *Biol. Trace Elem. Res.* **150**, 477–486 (2012). 23054864 10.1007/s12011-012-9510-x

[33] Boggs, D. R. & Joyce, R. A. The hematopoietic effects of lithium. *Semin Hematol.* **20**, 129–138 (1983). 6348956

[34] Boggs, D. R. & Joyce, R. A. Lithium effects on stem cells – advances in stem cell application in clinical medicine. *Adv Cell Sci Tissue Cul.* **2** (1), 14–24 (2018).

[35] Zhu, Z. et al. Lithium stimulates human bone marrow derived mesenchymal stem cell proliferation through GSK-3β-dependent β-catenin/Wnt pathway activation. *FEBS J.* **281**, 5371–5389 (2014). 25265417 10.1111/febs.13081

[36] Wang, Q. et al. Lithium, an anti-psychotic drug, greatly enhances the generation of induced pluripotent stem cells. *Cell. Res.* **21**, 1424–1435 (2011). 21727907 10.1038/cr.2011.108 PMC3193456

[37] Jin, S. Autophagy, mitochondrial quality control, and oncogenesis. *Autophagy* **2**, 80–84 (2006). 16874075 10.4161/auto.2.2.2460

[38] Nakatogawa, H., Suzuki, K., Kamada, Y. & Ohsumi, Y. Dynamics and diversity in autophagy mechanisms: lessons from yeast. *Nat. Rev. Mol. Cell. Biol.* **10**, 458–467 (2009). 19491929 10.1038/nrm2708

[39] Tidwell, T. R., Røsland, G. V., Tronstad, K. J., Søreide, K. & Hagland, H. R. Metabolic flux analysis of 3D spheroids reveals significant differences in glucose metabolism from matched 2D cultures of colorectal cancer and pancreatic ductal adenocarcinoma cell lines. *Cancer Metab.* **10**, 9 (2022). 35578327 10.1186/s40170-022-00285-w PMC9109327

[40] Weiswald, L. B., Bellet, D. & Dangles-Marie, V. Spherical cancer models in tumor biology. *Neoplasia N Y N*. **17**, 1–15 (2015). 10.1016/j.neo.2014.12.004 PMC4309685 25622895

[41] Sehgal, S. A. et al. Pharmacological progress of mitophagy regulation. *Curr. Neuropharmacol.* **21**, 1026–1041 (2023). 36918785 10.2174/1570159X21666230314140528 PMC10286582

[42] Wang, S. et al. The mitophagy pathway and its implications in human diseases. *Signal. Transduct. Target. Ther.* **8**, 304 (2023). 37582956 10.1038/s41392-023-01503-7 PMC10427715

